# Long-term neuroprotection of retinal ganglion cells by inhibiting caspase-2

**DOI:** 10.1038/cddiscovery.2016.44

**Published:** 2016-06-13

**Authors:** V Vigneswara, Z Ahmed

**Affiliations:** 1 Neurotrauma Research Group, Institute of Inflammation and Ageing, College of Medical and Dental Sciences, University of Birmingham, Birmingham, UK

Caspases are a family of cysteine-rich proteases that activate apoptotic pathways through their ‘initiator’ or ‘executioner’ pathways. Initiator caspases activate executioner caspase, such as caspase-2, -3, -6 and -7, which hydrolyze or cleave structural proteins in the cell leading to apoptosis. Caspase-2 is the most highly conserved caspase and uniquely fits into both initiator and executioner categories and as such partakes in a variety of apoptosis inducing signals including DNA damage, heat shock, endoplasmic reticulum and oxidative stress. Caspase-2-deficient neurons are resistant to apoptosis induced by beta-amyloid although it is involved in apoptosis of hippocampal neurons after ischemia and is expressed in ischemic retinae.^1^

The protection of retinal ganglion cells (RGC), particularly after trauma and numerous ocular pathologies such as glaucoma and ischemic optic neuropathy, is of particular interest to the ocular biology field. RGC loss, a hallmark of these diseases leads to vision loss and blindness.^2^ RGC are particularly susceptible and in the rat optic nerve transection/crush model in adult rats, RGC die rapidly and within 28 days, 90% of RGC are lost.^3^ As apoptosis accounts for the majority of RGC death, treatments that inhibit apoptosis afford limited neuroprotection, including microglial inhibitors, brain-derived neurotrophic factor, over-expression of Bcl-2 and caspase inhibitors.^4^ Caspase-3, -6, -8 and -9 are all regarded as important for RGC apoptosis, however, inhibition of these caspases, either alone or in specific combinations afforded protection of up to 60% of RGC.^5^ Furthermore, our observations that cleaved caspase-3 was absent in RGC and that little or no changes in cleaved caspase-6 and -7 were detected after transection of RGC axons by optic nerve crush (ONC) suggested that other caspases may have a part in RGC apoptosis.^3,6^

We previously reported that RGCs exclusively expressed activated (cleaved) caspase-2 after ONC in adult rats and confirmed these findings by employing an unbiased caspase trapping assay.^3,6^ We further showed that inhibition of caspase-2 by either a pharmacological inhibitor^6^ or a chemically modified synthetic short interfering RNA (siCASP2) significantly protected RGC from death, the latter protecting >95% of RGC from death for up to 21 days after ONC.^3,7^ Here, we demonstrate in retinal whole mounts, prepared after FluroGold backfilling of RGC,^3,7^ that intravitreal injection of siCASP2 every 8 days protected RGC from death at all radial quadrants from the optic disc including the central, mid-periphery and outer quadrants, with >95% protection of RGC from death over the entire retina at 12 weeks after ONC (Supplementary Figure S1), compared with intact controls (2170±200 in siCASP2 treated eyes *versus* 2250±230 RGC/mm^2^ in intact controls). Only a small proportion of RGC were present in retinal whole mounts from control scrambled siRNA-(siCNL^3^) treated eyes (150±130 RGC/mm^2^). Our results suggest that neuroprotection by siCASP2 is extremely efficient throughout the entire retina and that this is an unprecedented level of neuroprotection. To the best of our knowledge, our results far exceed those of any others published in the literature. Our results affirm that caspase-2 is the main caspase responsible for the death of RGC after direct trauma. This bodes well for siCASP2, otherwise known as QPI-1007, currently recruiting patients in a worldwide Phase III trial that is being initiated by Quark Pharmaceuticals Inc (USA) (http://quarkpharma.com/?p=12241).
