# Supplementary figures and images for: Long-term neuroprotection of retinal ganglion cells by inhibiting caspase-2
Source: Cell Death Discov. 2016 Jun 13;2:16044–. doi: 10.1038/cddiscovery.2016.44 (PMC4979513; doi:10.1038/cddiscovery.2016.44)

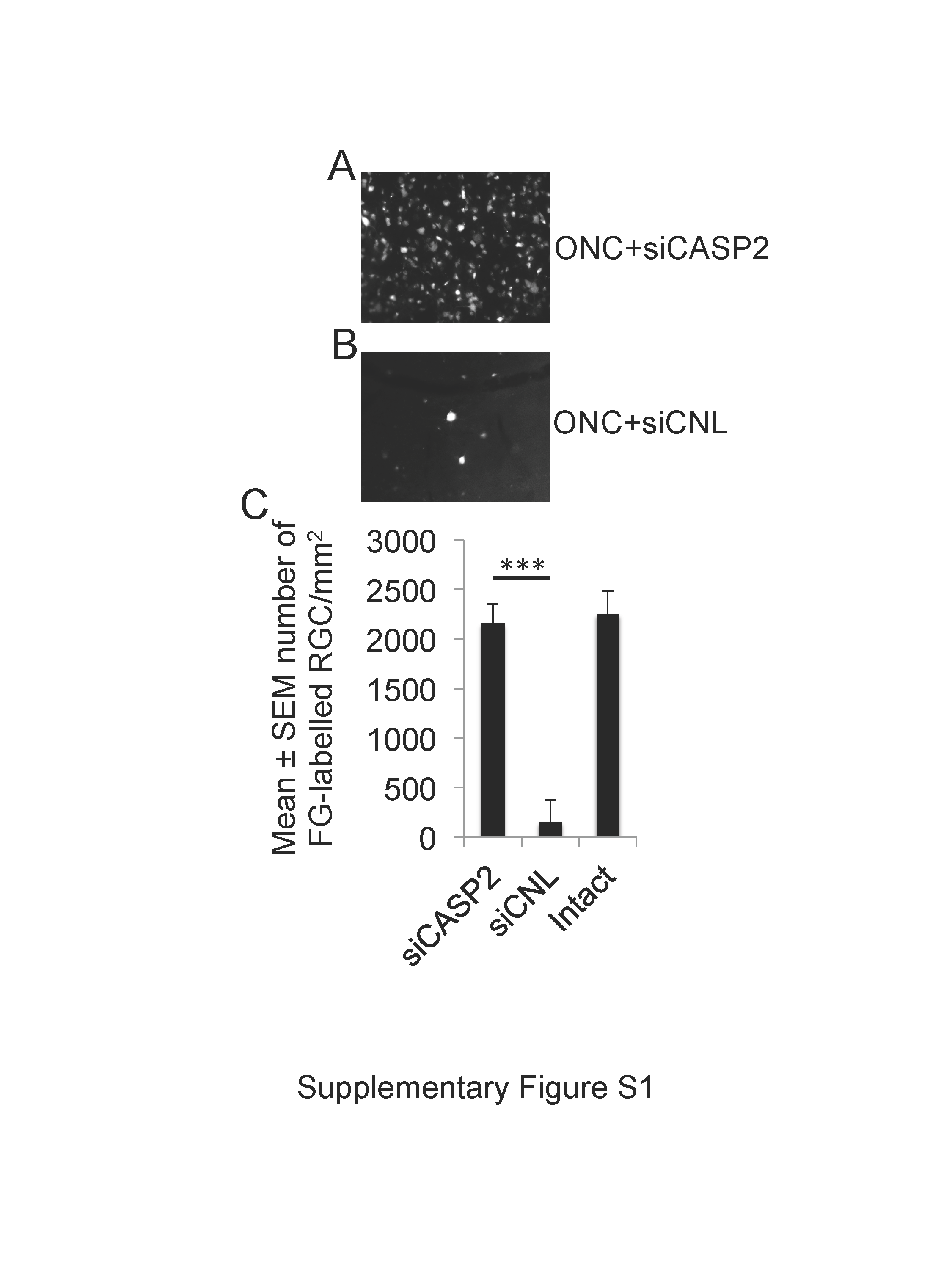

Supplement: Supplementary Figure S1 [file cddiscovery201644-s1.tiff]
