# Supplementary material for: Long-term neuroprotection of retinal ganglion cells by inhibiting caspase-2
Source: Cell Death Discov. 2016 Jun 13;2:16044–. doi: 10.1038/cddiscovery.2016.44 (PMC4979513; doi:10.1038/cddiscovery.2016.44)
Supplement: Supplementary Information [file cddiscovery201644-s2.doc]

**Materials and Methods**

All animals procedures are licensed by the UK Home Office and approved by the University of Birmingham Animal Welfare and Ethical Review Board. Adult rat optic nerves (n = 12/treatment) were crushed biliaterally and treated with either siCASP2 or siCNL (a control scrambled siRNA) and allowed to survive for 12 weeks. Two days before killing animals, optic nerves were injected with 2μl FluoroGold (FG) and retinae harvested for retinal wholemounts. FG-labelled RGC were visualised using an Axioplan fluorescent microscope equipped with an Axiocan HRc and Axiovision software. Images were captured from the inner, mid-periphery and the outer quadrant of the retinae (16 images/wholemount) and FG-labelled RGC were counting using the automated counting facility in Image J (NIH, USA).

**Supplementary Figure S1:** Suppression of caspase-2 promotes long-term RGC survival. (A) Representative FG-labelled RGC mid-periphery of retinal whole mounts after treatment with ONC+siCASP2 and (B) ONC+siCNL. (C) Quantification of the mean number of FG-labelled RGC across the whole retina after treatment with siCASP2, siCNL and in intact control retinae.
